# Thunderclap headache revealing dural tears with symptomatic intracranial hypotension: Report of two cases

**DOI:** 10.3389/fneur.2023.1132793

**Published:** 2023-02-23

**Authors:** Dana Antonescu-Ghelmez, Ioana Butnariu, Florian Antonescu, Cristina Maier, Adriana Moraru, Amanda Ioana Bucur, Daniela Nicoleta Anghel, Sorin Tuţă

**Affiliations:** ^1^Department of Neurology, “Carol Davila” University of Medicine and Pharmacy, Bucharest, Romania; ^2^Department of Neurology, National Institute of Neurology and Neurovascular Diseases, “Carol Davila” University of Medicine and Pharmacy, Bucharest, Romania; ^3^MedInst Romanian-German Diagnostic Center, Bucharest, Romania

**Keywords:** post-traumatic dural tear, CSF leakage, intracranial hypotension, sacral fracture, sacral osseous fragment, severe headache, thunderclap headache, case report

## Abstract

Cerebrospinal fluid (CSF) leakage is considered the cause of spontaneous intracranial hypotension (SIH), an important etiology for new daily persistent headaches and a potentially life-threatening condition. Minor traumatic events rarely lead to CSF leakage, contrasting with iatrogenic interventions such as a lumbar puncture or spinal surgery, which are commonly complicated by dural tears. Most meningeal lesions are found in the cervicothoracic region, followed by the thoracic region, and rarely in the lumbar region, and extremely rarely in the sacral region. We describe two patients admitted to our hospital for severe headaches aggravated in the orthostatic position, with a recent history of minor trauma and sustained physical effort, respectively. In the first case, a bone fragment pierced an incidental congenital meningocele creating a dural fistula. An extensive extradural CSF collection, spanning the cervicothoracic region (C4–T10), was described in the second case. In both patients, the clinical evolution was favorable under conservative treatment.

## Introduction

Tears of the spinal dural membrane, rupture of a meningeal diverticulum, and development of CSF-venous fistulas are the major sources of cerebrospinal fluid leaks (1). Dural tears may occur spontaneously or after deliberate or accidental disruption of the meninges (2, 3). Meningeal diverticula, also known as *meningoceles*, which may be found at single or at multiple levels simultaneously (4, 5), are frequently associated with connective tissue abnormalities, and develop spontaneously or after the healing of a dural tear (6). CSF leakage is a known cause of spontaneous intracranial hypotension (SIH), an under-diagnosed although not so rare syndrome characterized by a classical triad of low cerebrospinal fluid pressure, orthostatic headache, and brain “sag” with diffuse pachymeningeal enhancement on magnetic resonance imaging (MRI) (7, 8). We present two cases of intracranial hypotension with onset after a minor fall and, sustained carrying effort, respectively.

## Case presentation

### Patient 1

A 37-year-old previously healthy female patient was admitted to our hospital for severe orthostatic headaches with onset 5 days prior, after a seemingly innocuous fall on the buttocks during a ballet lesson, which she was practicing as a hobby. No history of direct head trauma or whiplash was reported. She reported no back pain. The headache was holocephalic, exacerbated by the orthostatic position, slightly relieved by clinostatism, and it was not associated with neck stiffness, fever, nausea, or vomiting. The physical exam was normal, without sensory or motor deficits, and without incontinence. A CT scan of the brain performed at admission was unremarkable.

**Figure 1:**
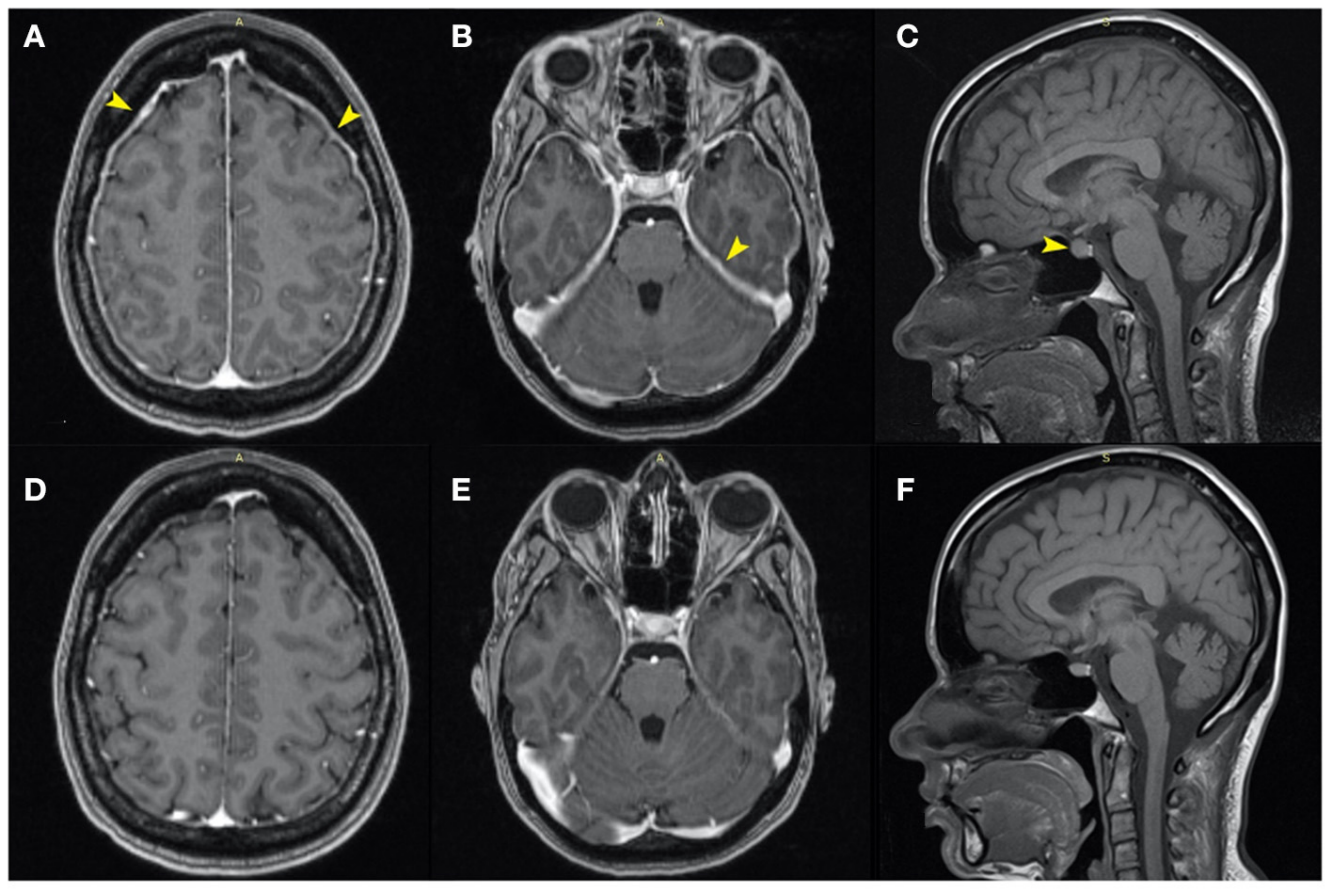
Axial MRI contrast-enhanced T1 images: similar sections at the moment of diagnosis **(A–C)**, and, respectively, 5 months later **(D–F)**. Initially, the MRI revealed diffuse, relatively uniform thickening and enhancement of the pachymeninges and tentorium, without pathological enhancement [**(A, B)**—yellow arrows] and an enlarged pituitary gland with a cranially convex superior border [**(C)**—yellow arrow]. Also, the dural venous sinuses and cortical veins were initially slightly engorged. Five months later the images showed normalization of the pachymeninges **(D, E)** and a reduction of the craniocaudal diameter of the hypophysis **(F)**.

Considering the sensitivity to changes in position, a characteristic element of SIH, a contrast-enhanced MRI of the brain was also performed (Figures 1A–C). The MRI revealed diffuse, relatively uniform thickening and enhancement of pachymeninges, without pathological leptomeningeal enhancement (Figures 1A, B), an enlarged pituitary gland with a cranially convex superior border (Figure 1C) and slightly engorged dural venous sinuses and cortical veins, all findings suggestive of intracranial hypotension syndrome. Otherwise, the brain examination was normal.

**Figure 2:**
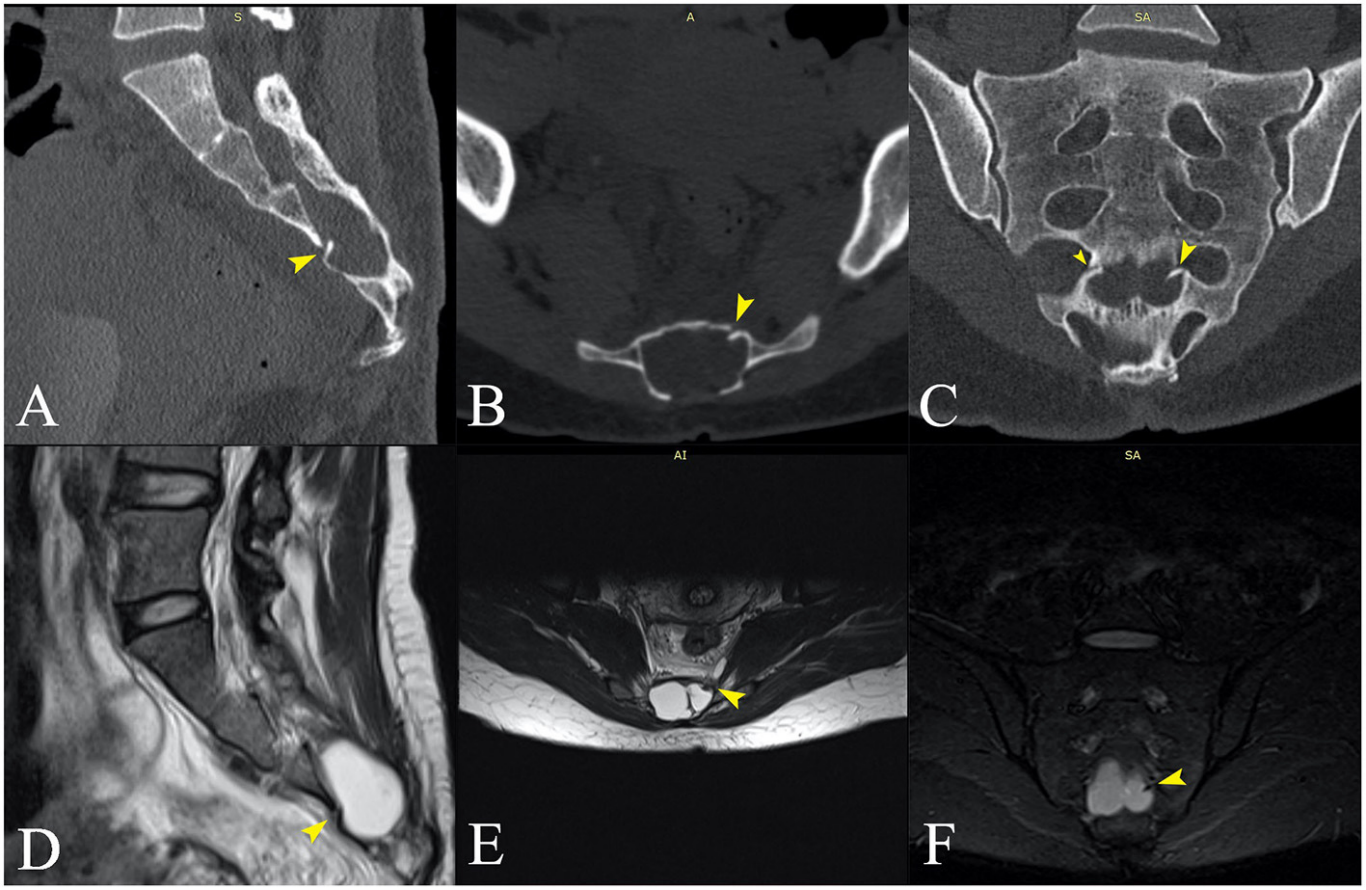
**(A–C)** Sagittal, axial, and coronal computed tomography, and **(D–F)** MRI images at approximately the same anatomical plane of section. **(D–F)** Voluminous meningoceles visible on the MRI images. **(B)** The posterior dysraphism is clearly visible on the CT transversal section. **(C)** The fracture line is best visualized in the coronal plane (small and large yellow arrows), with a bone fragment protruding into the sacral canal on the left side [**(A–F)**—large yellow arrows]. **(D, E)** The bone fragment clearly imprints the left sacral meningocele and appears to pierce it **(F)**.

We followed up with a contrast-enhanced MRI of the whole spine to search for a possible dural defect. The cervicothoracic spinal cord morphology and signal were normal. However, the examination revealed anterior and posterior epidural “banded” enhancement of the whole spine more evident at C2–C3 (with a maximum thickness of 6 mm) in the continuity of the infratentorial pachymeningeal thickening, probably reflecting engorgement of epidural venous plexuses. The medullary cone was in a low position (L3), and the terminal filum was thickened and attached posteriorly at L4–L5. There was a wide posterior S3–S4 dysraphism, and bilateral S3 bulky meningocele (32/22/22.5 mm on the right side and 27/16/21 mm on the left side) occupied the sacral canal (Figures 2D, E) exerting significant mass effect with pressure atrophy of the S3, S4 bodies and of posterior vertebral elements. Probably incidentally, two periradicular arachnoid cysts were present on the left S2 and S4 roots (Figure 4). The anterior sacral wall seemed fractured at the S4 level, with a small left anterolateral bone fragment that appeared to protrude internally (Figure 2F). Dural discontinuity of the anteroinferior wall of the left meningocele toward the presacral space was observed as the above-mentioned bone fragment seemed to pierce it. Considering the malformed osseous aspects, the suspected sacral fracture and limited MRI resolution in regard to bone, a computed tomography of the spine was also performed, confirming the S4 fracture extending between both anterior holes with a displaced bony fragment (5/2 mm) protruding into the sacral canal (Figures 2A–C).

After 7 days of symptomatic analgesic treatment, intravenous fluid therapy, and prolonged supine position, the headaches improved significantly. At the 5 months follow-up, the patient had no headache and no neurologic deficits. The brain MRI also showed marked improvement, with the disappearance of the thickening of the pachymeninges (Figures 1D, E) and with a slightly reduced size of the pituitary gland (Figure 1F).

### Patient 2

An overweight 38-year-old man, a smoker, presented with frontal and temporal headaches with sudden onset the previous day. The headaches were associated with nausea and vomiting and were relieved by the supine position. After several hours, severe cervical pain was added to the symptomatology. The patient had not suffered any obvious trauma and had no medical history. Still, a few days before the onset of the symptoms, he had made an intense physical effort on a mountain hike when he carried his daughter, weighing about 12 kg, on his shoulders for several hours a day for 2 days in a row. No neck stiffness, fever, or neurological deficits were found during the clinical examination. A CT scan and a DWI-MRI sequence of the brain were performed at the admission, and both were normal. Given the sudden onset and the associated emetic syndrome, a lumbar puncture was performed to rule out a possible subarachnoid hemorrhage. The opening pressure was within normal range, the CSF was clear, and the laboratory results were unremarkable.

**Figure 3:**
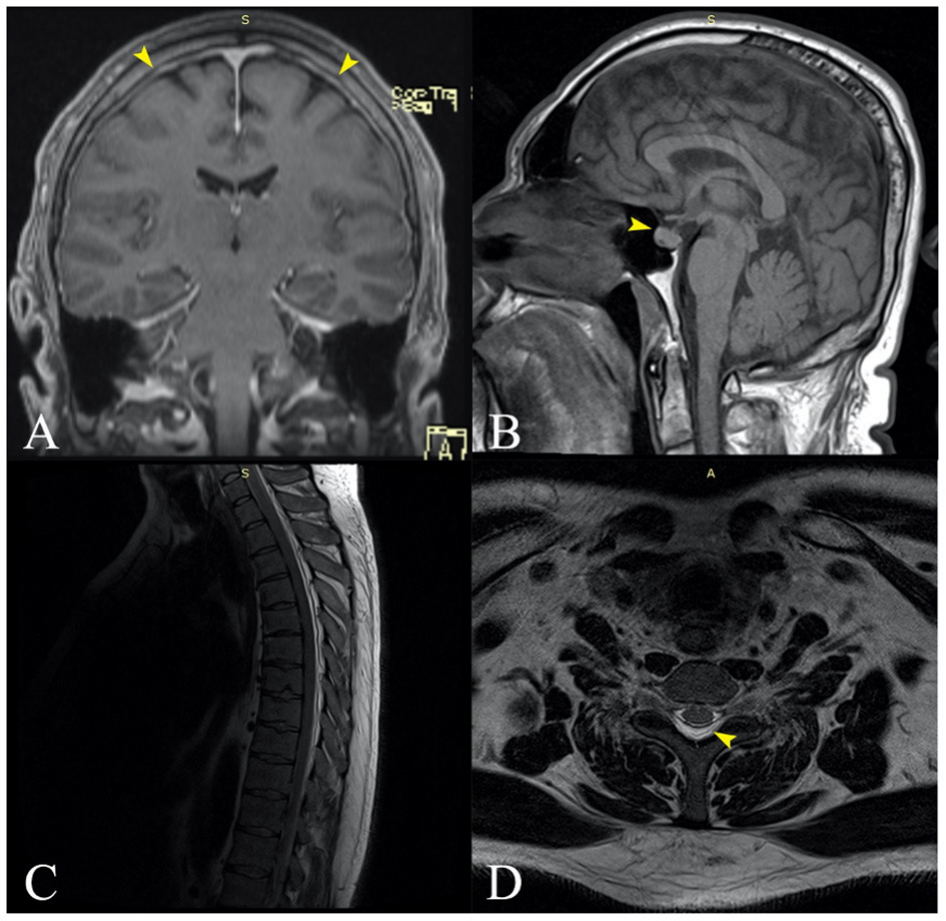
**(A)** Coronal MRI contrast-enhanced T1 image revealing slight diffuse thickening of the cerebellar tentorium and pericerebral pachymeninges, especially bilateral paramedian parietal [**(A)**—yellow arrows]. **(B)** Sagittal MRI contrast-enhanced T1 showing an enlarged pituitary gland with superior border cranially convex [**(B)**—yellow arrow]. **(C)** Sagittal spine MRI showing a posterior cervicothoracic epidural collection with fluid signal, extended craniocaudal from the C4 to T10, with a maximum thickness of about 5 mm in the thoracic region. The thoracic spinal cord is anteriorly displaced but not compressed. **(D)** Transversal MRI section of the thoracic spine (T6) showing the posterior epidural collection that displaces the spinal content anteriorly [**(D)**—yellow arrow].

Next, we ordered a contrast-enhanced MRI of the brain, which revealed mild diffuse cortical vein engorgement visible on SWI, minimal diffuse thickening of the cerebellar tentorium and pericerebral pachymeninges (especially bilateral paramedian parietal) (Figure 3A), and an enlarged pituitary gland with superior border cranially convex (Figure 3B), all of which suggested intracranial hypotension. Finally, the patient underwent a spine MRI that showed a posterior cervicothoracic epidural collection with fluid signals similar to the CSF (Figures 3C, D), extending craniocaudally from the right side of C4 to T10, with a maximum thickness of about 5 mm in the thoracic region. The thoracic spinal cord was anteriorly displaced but not compressed. Also, we observed engorgement of the posterior epidural venous plexuses, supporting the diagnosis of intracranial hypotension syndrome. Under conservative treatment with non-steroidal anti-inflammatory and antiemetic drugs, the symptomatology gradually improved. The patient was discharged after 10 days without any complaints or neurological deficits. He did not attend his scheduled follow-up.

## Discussion

Low CSF pressure syndromes are classified as primary (or spontaneous) and secondary (9). A CSF leak is described as spontaneous when there is no proceeding history of a major traumatic event or spinal medical procedures (7, 8). Spontaneous CSF leaks have a multifactorial etiology, with three main production mechanisms: meningeal diverticula leakage, spinal ventral dural tears, and cerebrospinal fluid–venous fistula (10). The meningeal diverticula develop along a nerve root sleeve, especially in the thoracic or upper lumbar spine, and may involve large meningeal tears with high-flow CSF leaks or slow-flow leaks facilitated by Valsalva-like maneuvers. Ventral dural tears are most commonly caused by osteophytes or calcified intervertebral disks that incise the dura, producing a longitudinal tear. They are associated with high-flow CSF passage to the epidural spaces, and the protrusion of the spur into the tear may prevent spontaneous healing, requiring surgical treatment. In the cerebrospinal fluid–venous fistula, there is a direct connection between the spinal subarachnoid space and a segmental spinal vein, with rapid loss of CSF following the pressure gradient, which is higher in CSF than in the venous segment. Dynamic CT myelography can detect the pathological filling of the vertebral venous network from the CSF-injected contrast media. Spontaneous improvement is possible in low-flow spinal CSF leakage, while in high-flow and cases with protruding osseous spurs, interventional treatment are often needed (epidural targeted blood patch or neurosurgical closure of the defect) (11).

In about one-third of the patients, a history of mild trauma, such as coughing, sneezing, or lifting can be noted (5, 8). Two-thirds of the patients diagnosed with spontaneous CSF leaks and meningeal diverticula suffer from a generalized connective tissue disorder (5).

Secondary CSF leaks are caused by invasive procedures, such as spinal tap, myelography, spinal surgery, pneumonectomy, or by major traumatic events (9). They can also be associated with other underlying medical conditions, such as degenerative pathology of the spine, arteriosclerosis, or dehydration (2, 10).

**Figure 4:**
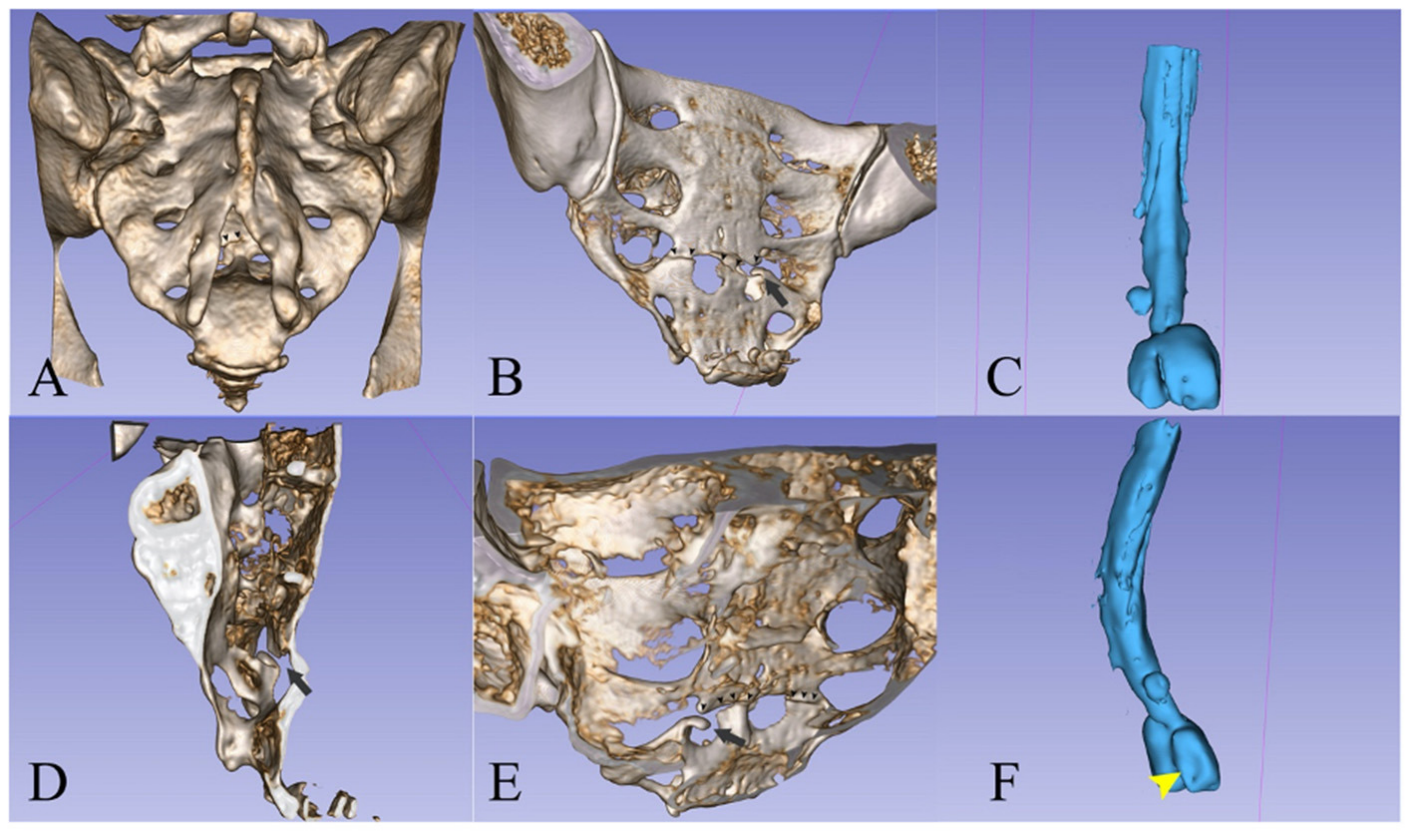
**(A, B, D, E)** 3D reconstruction (13) of the sacrum from the CT scan images. **(C, F)** A spinal canal mold from the CSF signal on the MRI images. The left S2 radicular cyst is visible above the meningoceles. **(A)** Posterior view showing the dysraphism at the level of the bone. [**(A)**—small arrowheads] The fracture line is partially visible. [**(B)**—small arrowheads] Posterior oblique view of the pelvic surface of the sacrum showing the transverse fracture at S4 and [**(B)**—black arrow] the bone fragment that protrudes into the spinal canal. **(C)** The spinal canal ends with two large meningoceles at the S3 level. **(D)** Interior view of the sacral canal from the midline, the black arrow points to the protruding bone fragment. [**(E)**—small arrowheads] Interior view of the posterior wall of the sacral canal, showing the line of fracture and [**(E)**—black arrow] the protruding fragment. **(F)** Oblique posterior view showing the imprint of the fractured bone fragment on the left meningocele.

The principal consequence of CSF leaks is SIH. This syndrome has an incidence of 2–5/100,000/year with a peak age of 30–50 years and female predominance. It is characterized by the classic triad of low CSF pressure, orthostatic headache, and brain “sag” with a diffuse pachymeningeal MRI enhancement (7, 12). In our first case, the patient suffered a minor accident by falling during a ballet lesson. The patient had an undiagnosed malformation of the sacral spine (wide posterior S3–S4 dysraphism and bilateral S3 meningoceles) which, combined with the minor trauma, proved causal. The fall produced a transverse S4 fracture, from which an osseous fragment seems to have pierced the dura (Figure 4). Sacral fractures are uncommon injuries that most often result from high-energy trauma such as motor vehicle accidents and falls from an elevation (14). Only 5% of sacral fractures occur in isolation, 45% occur with a concomitant pelvic ring injury, and up to 50% are associated with neurological injuries (14, 15). Low transverse fractures, at the level of S3 or below, typically result after a fall onto the buttocks, as was the case in our patient. Isolated fractures in the lumbosacral region may rarely be complicated with dural injury, causing CSF leakage and formation of a traumatic pseudomeningocele (16–18).

The second patient carried his daughter on his shoulders for several hours during a hike. The exact location of leakage was not identified, but the most probable mechanism remains a tear of the dural sheath of nerve roots at an anatomically weak point in the cervical region or at the cervicothoracic junction. As the evolution was spontaneously favorable, a myelography was not performed. Neither patient had history or clinical signs of any connective tissue disorder.

The most susceptible regions for spontaneous dural leakage are the cervical and cervicothoracic junction, but the thoracic, lumbar, and sacral regions, and even the vestibular system, the cribriform plate, and the pituitary fossa, can be involved (8, 10). Once the dural tear is produced, the CSF may outflow into the extradural tissues, where it is absorbed in the initial phase after the trauma, but if the opening persists, the capacity of absorption is exceeded, CFS accumulates, and a non-absorbing membrane may gradually develop, forming a cyst (19). The time period between the formation of the fluid collection and its diagnosis varies from months to years. The volume of the CSF leak may vary from a small amount detectable only while performing the Valsalva maneuver to a large amount dispersing into the paraspinal soft tissues (5).

The clinical hallmark of SIH is the positional headache that occurs or worsens most often within the first 15 min of assuming an upright posture (20). Sometimes this delay may last even several hours. The pain is generally relieved within 30 min after lying down (20). Its onset is usually gradual with a maximum intensity reached after several minutes, but, in 15% of cases, a “thunderclap” type of headache can occur (7, 20, 21). The headache may be diffuse holocephalic, frontal, or temporal, but typically it is localized in the back of the head or base of the skull, rarely unilaterally (5, 22). It can be throbbing, and it is often characterized as a “pulling sensation” from the back of the head to the neck (22). The severity of the pain ranges from mild to incapacitating, with sometimes patients being unable to assume an upright position (5). Although orthostatic headache is typical in SIH, the postural aspect may be absent or it can diminish with the passing of time if the CSF leak remains untreated. Moreover, many different headache patterns have been described, such as exertional headaches, “second-half-of-the-day” headaches, cough headaches, and paradoxical headaches occurring or worsening when lying down. The latter is associated with the rebound of intracranial pressure after dural fistula closure (5, 23).

Apart from the headaches, other manifestations may occur in SIH, the clinical spectrum being wide and probably not yet fully defined (22, 23). The severity of the manifestations is also variable: some patients are minimally affected, while others are seriously disabled while upright (22). The most common features reported are neck pain or stiffness, nausea, and vomiting (5, 10). The stretching of cranial nerves following the downward displacement of the brain may cause, in <10% of patients, visual blurring or visual field deficits, diplopia, facial pain or numbness, facial weakness or spasm, dysgeusia, and, in 10–50% of cases, changes in hearing (“echoing” or “being underwater”), associating tinnitus, or imbalance (5, 10). If the downward displacement is important, it may lead to cerebellar tonsillar herniation, brainstem compression with depression of the vital centers, altered mental status, and coma (5, 24). Cognitive deficits ranging from minimal memory loss to signs typical of a major cognitive disorder may complicate SIH (5, 22).

In our cases, the headache was the central manifestation, there were no neurologic deficits, and only the male patient presented cervical pain.

MRI has marked a new era in the understanding of SIH and its diagnosis (7, 22). It is now considered the method of choice in the initial evaluation when SIH is suspected, being the most sensitive form of investigation. The presence and the severity of MRI abnormalities are variable, and a normal MRI, as happens in 20% of cases, does not exclude SIH (5, 22).

Typically, the brain MRI in SIH may reveal diffuse, non-nodular enhancement of the pachymeninges sparing the leptomeninges, subdural fluid collections, engorgement of venous structures, “sagging” of the brain, pituitary hyperemia, obliteration of the prepontine and perichiasmatic cisterns, and ventricular collapse (5, 7). Diffuse pachymeningeal enhancement is the first sign to appear, being identified in 73% of patients (25). The mechanisms are currently thought to be the dilatation of the dural veins and various grades of edema in the meningeal layers, external to the arachnodural junction, both secondary to the low CSF pressure (26).

The MRI findings may improve in days to weeks after effective specific treatment of a CSF leak (5). In conservatively managed cases, the enhancement may persist, but generally, it becomes less prevalent over time (27). This seems to occur not only in patients with favorable clinical evolution but also in some patients with persistent symptoms. It seems that in these cases, compensatory mechanisms manage to alleviate the effects of CSF volume depletion over time, and this results in variable improvement of the MRI signs, which does not necessarily translate to clinical improvement. CSF pressure in patients with SIH seems to increase over time, independent of the presence or absence of CT myelographically detectable CSF leakage, which also suggests that compensatory mechanisms develop over time (28).

It is not clear how soon the MRI signs appear after the onset, as the literature in this area is sparse, yet we assume it would be of the order of hours, as it would not take much longer for the dural veins to feel the effects of the low pressure. There are many published cases that document the MRI alterations in the first week (27) and we have also identified one with severe imaging findings in the first 48 h from onset of symptoms (29).

Both our patients responded to conservative measures and the follow-up imaging studies revealed significant improvements in one of the cases.

Additionally, when a CSF leak is suspected, a full spine imaging study must be performed (7). CT myelography is the investigation of choice, but non-invasive techniques, such as spinal MRI or MR myelography, are now advocated as first-line methods for the diagnosis of CSF leaks (22, 30). Spinal findings in SIH include dural enhancement, meningeal diverticula, extrathecal CSF collections, syringomyelia, and dilated epidural or intradural veins (5).

In selected cases, a lumbar puncture may also be performed. Generally, the CSF opening pressure under 60 mm H_2_O has diagnostic value, but CSF pressure in the normal range (7–20 cm H_2_0) is found in many patients. Occasionally, CSF pressures may be >20 cm H_2_0 despite an active CSF leak (28). This is the reason why the International Classification of Headache Disorders' (ICHD-3) major diagnostic criteria of intracranial hypotension headache include either low cerebrospinal fluid (CSF pressure < 60 mm H_2_O) or evidence of CSF leakage on imaging (31).

In our patients, lumbar puncture was performed only in the second case, as the acute nature of the pain raised the suspicion of a subarachnoid hemorrhage. The opening pressure was normal, but, as we mentioned above, this is known to occasionally occur, and it may explain the self-limited course and quick recovery (32). It is very probable that, as the epidural collection expanded, the pressure between CSF and the surrounding epidural space had balanced.

An interesting discussion arises referring to “thunderclap” headaches with normal imaging, normal CSF exam, and normal opening pressure, which, generally, remain categorized as idiopathic. This probably would have been the case in our second patient, in which cerebral signs of intracranial hypotension were faint, had we not visualized the extradural CSF collection. Our case suggests that micro-dural tears, which cannot be observed on current MRI protocols, could explain some cases of cryptogenic “thunderclap” headaches, and also that the site of the lesion may not be at the cranial level. We think it may be useful in selected cases to order a complete spinal MRI examination.

Management of intracranial hypotension depends on the severity of the signs and symptoms, on the presence of extradural CSF collections, and their size (33). Many patients (28%) respond to conservative measures, such as bed rest, adequate oral fluid intake, pain medication, caffeine, and the use of an abdominal binder (5, 8, 24, 25). However, if the CSF leak has a high flow, the symptoms may persist regardless of conservative treatment. In those cases, a non-targeted epidural blood patching is performed at the thoracolumbar junction, effective for 64% of patients (25). If unsuccessful, targeted CT-guided blood patches at the site of the leak, percutaneous placement of fibrin glue, or surgical procedures may be attempted (7, 24, 25). In Table 1, we review the treatment and outcome in recent cases of SIH (last 5 years) reported in the literature.

**Table 1:** Treatment and outcome in recent cases of SIH (last 5 years) reported in the literature.

**References**	**NOC**	**Age (y)**	**G**	**Conservative treatment**	**EBP**	**Repeated EBP**	**Surgical treatment**	**Clinical follow-up**	**Imaging follow up**
Arshad et al. (34)	1	49	M	N/A	No	No	“Snowman” pledget technique	Improvement	N/A
Barral et al. (35)	1	26	F	BR, H	No	No	None	Improvement	N/A
Casanova et al. (36)	1	79	F	N/A	No	No	Duraplasty	Complete recovery	Brain CT: Complete resolution of the SDH
Choi et al. (37)	1	60	M	BR, H, A	Yes (C4–C5) FG	Yes	Burr hole drainage	Improvement	MR-M: Decreased size of C1–C2 CSF collection
Hughes and Chavez (38)	1	45	F	Caffeine	Yes	No	None	Complete recovery	N/A
Jafari et al. (39)	1	39	F	BR, caffeine	Yes	Yes (twice)	None	Complete recovery	N/A
Kanao-Kanda et al. (40)	1	46	M	N/A	With contrast agent, FG	No	None	Complete recovery	Spine MRI: Complete resolution of CSF leakage
Lee et al. (41)	1	45	M	BR, A	Yes(L4–L5)	Yes (T1–T2)	None	Complete recovery	Brain CT: Improvement
Masourou et al. (42)	3	53	M	N/A	Yes (L2–L3)	No	None	Complete recovery	Brain Gd + MRI improvement (SDH in remission, DPE remitted)
		38	M	H	Yes (L1–L2)	No	None	Complete recovery	Spine MRI: Improvement
		44	F	No	Yes (T11–T12)	No	None	Improvement	Spine MRI: Improvement
Moriyama and Ishikawa (43)	1	43	F	Yes	Yes (C1–C2) FG	No	None	Complete recovery	Spine MRI: Complete resolution of CSF leakage
Parra et al. (44)	1	34	M	A, gabapentin and amitriptyline, yoga	Yes (C7–T1)	Yes (C6–C7) + (L2–L3)	None	Improvement	N/A
Reihani et al. (45)	1	32	F	Caffeine, a high-salt diet, H, BR, A	No	No	None	Improvement	Brain Gd + MRI: Almost no abnormal findings
Shekhawat et al. (46)	1	45	M	BR, H, A, caffeine	Yes (C7–T1)	Yes (C7–T1) CT-guided	None	Complete recovery	Brain Gd + MRI brain: improvement
Shimizu et al. (47)	1	16	M	BR, H	No	No	SDH drainage.	Complete recovery	Brain CT: No change in the volume of the AC and no recurrence of SDH.
Sobczyk et al. (48)	1	28	M	No	No	No	C2–C3 and partial C4 laminectomy, epidural space revision and dural plastic surgery with Tachosil and Tissel fibrin glue	Complete recovery	Brain Gd + MRI + CT: Improvement
Sugiyama et al. (49)	1	53	M	No	Yes (L2–L3)	No	None	Improvement	Brain Gd + MRI: Improvement
Tonello et al. (50)	1	38	M	BR, H, steroids	No	No	None	Complete recovery	Brain + Spine Gd + MRI: Improvement (except for localized PE)
Zabek and Turek (51)	1	37	F	BR, H, steroids	Yes (CT guidance)	No	None	Complete recovery	Brain Gd + MRI: Complete resolution
Cerulli Irelli et al. (52)	1	42	F	BR, H	Yes	No	None	Improvement	Brain Gd + MRI: Complete resolution
Cochran et al. (53)	1	64	F	No	No	No	T7 duraplasty. Repeated after 3–4 m	Improvement	Brain Gd + MRI: Complete resolution
Ghosh et al. (54)	7	12	F	N/A	Yes	Yes	None	Improvement	N/A
		11	M	N/A	Yes	Yes	None	Improvement	N/A
		18	F	N/A	Yes	Yes	None	Improvement	N/A
		17	F	N/A	Yes	Yes	None	Improvement	N/A
		20	F	N/A	Yes	Yes	None	Improvement	N/A
		15	F	N/A	Yes	Yes	None	Improvement	N/A
		17	F	N/A	Yes	Yes	None	Improvement	N/A
Kumar et al. (55)	1	37	F	BR, H, theophylline	Yes	No	None	Complete recovery	N/A
Liu et al. (56)	1	28	M	Yes	Yes (T3–4/T2–3)	Yes (5 times)	Burr hole drainage	Improvement	Brain Gd + MRI: SDH remitted; Spine Gd + MRI: CSF leakages remitted
Nisson et al. (57)	1	2	F	N/A	Yes (T11–12)	Yes (twice)	T5 laminectomy	Improvement	N/A
Pensato et al. (58)	1	74	M	No	Yes	No	None	Improvement	Serial brain Gd + MRI: Improvement
Podkovik et al. (59)	1	46	M	N/A	Yes	Yes (multiple times)	SDH drainage	Mild improvement	CT: SDH recurrence
Sajjadi et al. (29)	1	37	M	BR, steroids	Yes (T12–L1)	No	None	Complete recovery	N/A
Jensen et al. (60)	1	42	M	No	Yes	Yes (L3–L4) CT guidance	None	Improvement	N/A
Turnbull and Morreale (61)	1	59	F	H, A	Yes	Yes (twice)	Decompressive suboccipital craniectomy with C1 laminectomy and duraplasty; Bilateral T3–T6 exploratory laminectomy	Complete recovery	Gd + MRI: Complete recovery
Villani et al. (62)	1	30	F	N/A	Yes	Yes (three times)	None	Improvement	Spine MRI: IMPROVEMENT
Wei et al. (63)	1	67	M	BR, hydration,	Yes (T2–T3)	Yes (twice)	None	Improvement	CT-M: No evident CSF leakage
Akbar et al. (64)	1	25	M	A	Yes (C4–C5) FG	No	None	Complete recovery	N/A
Akiba et al. (65)	1	58	M	BR, H, A	Yes (C1–C2) FG	No	Burr-hole drainage of the SDH + blood patch + Laminoplasty C3–C4	Complete recovery	Spine imaging: Complete resolution
Arai et al. (66)	2	52	F	N/A	Yes (T2–T3)	No	SDH drainage; T11–12 laminectomy; duraplasty	Improvement	N/A
		61	M	N/A	Yes (T11–T13)	No	T1–T2 DM, duraplasty	Improvement	N/A
Arumugam et al. (67)	2	40	F	N/A	Yes (L4–L5)	No	None	Complete recovery	Brain imaging: Complete resolution
		35	M	N/A	Yes (L4–L5)	No	None	Complete recovery	N/A
Chung et al. (68)	1	50	M	No	Yes + intrathecal saline infusion	Yes (twice)	None	Complete recovery	Brain CT: Improvement
Ferrante et al. (69)	1	16	F	BR, H	EPB + fibrin glue	Yes	None	Complete recovery	Brain and spine MRI: Complete resolution
Ferrante et al. (70)	1	68	M	BR, H	No	No	None	Complete recovery	Brain Gd + MRI: Pneumocephalus remission
Ferrante et al. (71)	1	35	F	No	Yes	No	None	Complete recovery	N/A
Hatano et al. (72)	1	60	M	BR, H	Multi-level	No	None	Complete recovery	Brain CT: SDH resolution
Mandal et al. (73)	1	63	M	Caffeine	Yes	Yes	Ligature of CSF venous fistula, duraplasty	Improvement	Brain IRM: Improvement
Mirchi et al. (74)	1	12	M	N/A	Scheduled for multi-level	N/A	N/A	N/A	N/A
Podkovik et al. (75)	1	46	M	No	Yes (T12–L1)	Yes (T12–L1)	SDH drainage	Mild improvement	Brain CT: SDH worsening
Pollard and Pollard (76)	1	24	M	Caffeine	Yes	No	None	Complete recovery	N/A
Shahab et al. (77)	1	46	F	Steroids, dihydroergotamine mesylate	Yes	Yes (twice)	C1 and T11–T12 laminectomy	Improvement	Brain Gd + MRI: Complete resolution
Shim and Park (78)	1	60	F	No	Yes (C7–T1)	No	3 × craniotomy, cranioplasty	Complete recovery	Brain Gd + MRI: Complete resolution
Uchigami et al. (79)	1	51	M	BR, H	Yes (lumbar)	No	Burr hole drainage	Complete recovery	CT: SDH resolution
Ueberschaer et al. (80)	1	50	F	Caffeine	No	No	Spur removal and duraplasty	Complete recovery	Brain and spine MRI: Complete resolution
Videira et al. (81)	1	55	M	Yes	No	No	None	Complete recovery	Brain and spine MRI: Complete resolution
Yamamoto et al. (82)	1	52	F	No	Yes (T1–T2 and T12–L1)	No	None	Complete recovery	Brain Gd + MRI: CVT resolution
Yokoi et al. (83)	1	14	F	No	No	No	T6–T11 fusion, duraplasty	Improvement	Brain CT: Improvement
Zou et al. (84)	3	49	M	Reinforced restriction of physical activity	No	No	None	Complete recovery	Brain CT: SDH complete resolution
		53	M	Reinforced restriction of physical activity	No	No	None	Complete recovery	Brain CT: SDH complete resolution
		53	F	Reinforced restriction of physical activity	No	No	None	Complete recovery	Brain CT: SDH complete resolution
Bakan et al. (85)	1	32	F	N/A (2M)	Yes (L4–L5) FG	No	None	Complete recovery	Brain Gd + MRI: Complete resolution
Cohen-Addad et al. (86)	1	53	F	No	Yes	No	None	Complete recovery	Brain Gd + MRI: Improvement
Cultrera et al. (87)	2	40	F	No	Yes	No	None	Complete recovery	Brain imaging: Complete SDH resolution
		71	M	No	Yes (L3–L4)	No	SDH drainage.	Complete recovery	Brain imaging: Complete SDH resolution
Fiechter et al. (88)	1	46	F	BR, A	Yes	Yes (twice)	T12 laminoplasty	Complete recovery	N/A
Fujikawa and Saitoh (89)	1	41	F	No	Yes	No	None	Complete recovery	N/A
Han et al. (90)	1	30	M	No	Yes (C2–C3)	No	T3–4 laminectomy, AC removed	Complete improvement	Spine MRI: Complete resolution
Karsidag et al. (91)	1	35	M	BR, caffeine, A, codeine	No	No	None	Improvement	Spine MRI: Regression of the CSF collection
Muram et al. (92)	1	34	M	BR, H, caffeine	Yes	Yes (C7–T1)	L2 hemilaminectomy, duraplasty, SDH drainage	Complete recovery	Brain MRI: Complete resolution
Radhakrishnan et al. (93)	1	16	F	Steroids	No	No	None	Complete recovery	Brain Gd + MRI: Complete resolution
Rajesh et al. (94)	1	32	M	No	Yes	No	None	Complete recovery	N/A
Swallow and Doan (95)	1	56	M	No	Yes (L4–L5) FG	Yes (L2–L3)	None	Complete recovery	N/A
Uchino et al. (96)	1	36	M	BR, H	Yes (L1–L2) + intrathecal infusion	Yes (T6–T7)	None	Complete recovery	Brain Gd + MRI brain: Complete resolution
Williams et al. (97)	1	56	M	No	Yes	No	Burr hole drainages; C2 laminectomy, duraplasty	Complete recovery	N/A
Camilla et al. (98)	2	35	F	BR, steroids	No	No	None	Complete recovery	Brain Gd + MRI almost complete resolution
		66	F	BR, steroids	No	No	None	Improvement	Brain Gd + MRI: Improvement
Chai et al. (99)	1	54	F	No	No	No	C4–5 discectomy and fusion	Complete recovery	N/A
Fujii et al. (100)	1	33	F	yes	No	No	None	Complete recovery	Brain Gd + MRI: Improvement
Girão et al. (101)	3	49	M	Excluded—recent history of head trauma					
		35	M	BR, H, caffeine, A	Yes	No	None	Complete recovery	N/A
		35	F	BR, H, caffeine, A	No	No	None	N/A	N/A
Lee et al. (102)	1	43	F	BR, H	Yes (T9–10)	No	None	Complete recovery	Gd + MRI: Improvement
Ozyigit et al. (103)	1	70	F	Steroids	No	No	None	Improvement	Gd + MRI: Improvement
Sasikumar et al. (104)	2	64	M	N/A	Yes	Yes (twice)	N	Improvement	N/A
		80	F	N/A	No	No	None	Spontaneous resolution of symptoms	N/A
Staudt et al. (105)	1	55	M	N/A	Yes	Yes (six times), fibrin glue	Thoracic laminectomy for marsupialization of the largest cyst at the T10–11 level	Improvement	Brain Gd + MRI: Improvement
Takai and Taniguchi (106)	1	70	M	BR, H	Yes	No	Laminotomy, osteophyte removal, duraplasty SDH drainage	Improvement	Brain imaging: Complete SDH resolution
Tontisirin et al. (107)	1	57	M	BR, H, A	Yes (T9–10)	Yes (C7–T1)	Burr-hole drainage of the SDH + blood patch + Laminoplasty C3–C4	Complete recovery	Brain CT: SDH complete resolution
Turel et al. (108)	1	50	F	N/A	Yes	No	T10–11 laminectomy, duraplasty	Complete recovery	Spinal Gd + MRI: Complete resolution of CSF epidural collection

A, analgesics; AC, arachnoid cyst; BR, bed rest; CT-M, computer tomography myelography; CVT, cerebral venous thrombosis; DPE, diffuse pachymeningeal enhancement; DM, discogenic microspur; EBP, epidural blood patch; FG, fluoroscopic guidance; G, gender; Gd+, with gadolinium; H, hydration; MRI, magnetic resonance imaging; MR-M, magnetic resonance myelography; N/A, not available; NOC, number of cases; SDH, subdural hematoma.

Generally, the prognosis is excellent for patients with brain MRI abnormalities and focal spinal CSF leaks, with most of them achieving full recovery under first-line measures. However, those with normal initial MRI findings and diffuse multilevel spinal CSF leaks may have residual altered CSF dynamics or residual CSF leaks undetectable with current imaging techniques (5).

## Conclusion

SIH is a common but still underdiagnosed cause of new daily persistent headaches and may be a cause of “thunderclap” headaches. It can be spontaneous or triggered by seemingly minor traumatic events. A dural tear tends to occur at anatomically weak points, usually at the level of the cervicothoracic junction. Cerebral signs of IH must prompt an evaluation of the whole spine, as the leak may be at any level. Consideration must be given not to confuse an extradural collection with the point of the leakage, as this is not always the case. CT myelography remains the gold standard imaging exam to find the level of CSF leakage, while contrast cerebral MRI gives a solid proof of intracranial hypotension as the cause of headache. Fortunately, conservative measures are successful in most cases.

## Data availability statement

The original contributions presented in the study are included in the article/supplementary material, further inquiries can be directed to the corresponding author.

## Ethics statement

Ethical review and approval was not required for the study on human participants in accordance with the local legislation and institutional requirements. Written informed consent from the patients was obtained for the publication of these cases.

## Author contributions

DA-G, IB, FA, AM, AB, and DA: conceptualization. FA and CM: software and visualization. IB, DA-G, and FA: writing—original draft preparation. ST: writing—review and editing. All authors issued final approval for the version to be submitted.
